# Increased freedom of head movement mitigates stress and bacterial load in the airways of horses during transport

**DOI:** 10.3389/fvets.2024.1477653

**Published:** 2024-10-04

**Authors:** Yuji Takahashi, Hidekazu Niwa, Yusaku Ebisuda, Kazutaka Mukai, Toshinobu Yoshida, Sharanne Raidal, Barbara Padalino, Hajime Ohmura

**Affiliations:** ^1^Sports Science Division, Equine Research Institute, Japan Racing Association, Tochigi, Japan; ^2^Microbiology Division, Equine Research Institute, Japan Racing Association, Tochigi, Japan; ^3^School of Agricultural, Environmental and Veterinary Sciences, Charles Sturt University, Wagga Wagga, NSW, Australia; ^4^Department of Agricultural and Food Sciences, University of Bologna, Bologna, Italy; ^5^Faculty of Science and Engineering, Southern Cross University, Lismore, NSW, Australia; ^6^Miho Training Center, Japan Racing Association, Ibaraki, Japan

**Keywords:** tracheal wash, colony forming unit, microbiota, long-distance transport, welfare, equine

## Abstract

**Introduction:**

Protection of horse welfare during transport is crucial. The aim of this study was to determine the effect of head and neck restraint on behavior and airway bacteria.

**Methods:**

In a randomized crossover study, six healthy Thoroughbreds were transported by road for 22 h in an individual bay with tight head restraint (50 cm short-rope) or loose head restraint (95 cm long-rope). Behavioral parameters relating to head position, eating, and stress were monitored during transportation. Tracheal wash samples were obtained 6 days before and immediately after transport for bacterial culture and metagenomic analysis.

**Results and discussion:**

Compared to before transport, bacterial load (CFU/mL) after transport was significantly increased in the short-rope group (*p* = 0.04), whereas no changes were observed in the long-rope group. Transport significantly reduced Simpson index at phylum, class, order, and family levels in both groups (*p* < 0.001) of tracheal microbiota. In both groups, this reduction was associated with increases in the dominant members of relative abundance at phylum (Firmicutes: +24% in long-rope and +14% in short-rope), class (*Bacilli*: +20% in long-rope and +22% in short-rope) and family (*Streptococcaceae*: +22% in long-rope and +23% in short-rope) levels. Licking behavior during transportation with short-rope restraint was more frequent than in horses with long-rope restraint. These results suggest loose head restraint during transportation is likely to ameliorate stress and mitigate the associated increased bacterial load in the lower airways associated with transport. Further, head position during transportation is likely a more important determinant of airway hygiene and distress than duration of travel.

## Introduction

Transportation is a recognized stressor for horses. It has been associated with a variety of physiological responses including increased respiratory (1) and heart rates (2, 3), decreased parasympathetic nervous activity (3), increased plasma cortisol concentrations, and elevated white blood cell counts (4, 5). Adverse effects of transportation include injury (6, 7), gastrointestinal disorders (8–10), decreased virus neutralization titers against equine herpes virus (11), inflammation and bacterial contamination of the lower respiratory tract, and pneumonia (12–14). Ongoing research to minimize the adverse effects of transportation is essential in all equine disciplines.

Physical restraint is one of the key factors associated with transport-related problems (15). Horses may adopt an elevated head posture in order to maintain balance in a moving vehicle (16), or head elevation may be imposed on the horse by cross-tying and restraint in the transport environment. In horses confined in reproductive stocks, this posture has been associated with accumulated airway secretions and increased bacterial counts in tracheal wash samples and preferential multiplication of *β*-haemolytic *Streptococcus* spp. and *Pasteurellaceae*, bacteria implicated in the development of pleuropneumonia in horses (17, 18). In contrast, a lowered head position could facilitate tracheal mucociliary clearance mechanisms to prevent secretions from accumulating in the trachea (19). Subsequent studies have confirmed that similar changes occur in transported horses (19, 20). Intermittent relief of head position, by lowering the head for 30 min every 6 h, did not prevent the accumulation of inflammatory airway secretions or increased bacterial contamination of tracheal samples (18). Further studies are warranted to assess the effect of different types of head restraint on respiratory tract contamination during transportation.

Recent advances in sequencing and bioinformatics allow for more detailed quantitative analysis of the microbiota in specific organs. These next-generation sequencing techniques have recently been used to better understand the influence of factors such as physical condition and transport on the equine airway microbiota. Bond et al., demonstrated that there was a separation between bacterial communities in the lower respiratory tract of horses with inflammatory airway disease (IAD) and without any respiratory problems; six operational taxonomic units in the tracheal community had different abundance with disease status, with *Streptococcus* being increased in IAD horses (21). Padalino et al. (20) reported that 8-h transport induced a significant reduction in diversity of respiratory flora with *Pasteurellaceae* dominating the respiratory flora. *Streptococcaceae* and *Pasteurellaceae* are most commonly isolated from equine pneumonia cases associated with transport (22–24). Therefore, it is likely that restraint of head position during transport allows preferential multiplication of these organisms, with subsequent increased relative abundance of *Streptococcaceae* and *Pasteurellaceae* and reduced diversity of airway microbiota, compared to the condition where horses can move their head and neck to the lower position.

Transport may affect behavior of horses. During transport horses may show increased stress-related behaviors, including licking and chewing, and balance-related behaviors, depending on the transport conditions (25). The frequency of these behaviors is usually higher in short journeys than in long ones (26). The first hour of a journey is indeed considered the most stressful, when horses often show a repertoire of stress-related behaviors, such as pawing, pulling back, licking, and turning their head. The frequency of such behaviors then tends to decrease due to horses coping with the transport situation or due to tiredness (20). Improving transport conditions, for example by larger space allowance, more freedom of the head and neck movement, and traveling within the thermal comfort zone, has been recommended to optimize welfare of horses during transport (15).

The purpose of the present study was to investigate the effect of restricted head position during transport on behavior and bacterial contamination of the respiratory tract in horses. We hypothesized that head restriction during transport would be associated with increased bacterial contamination and decreased diversity, and would be associated with greater stress responses.

## Materials and methods

The study was approved by the Animal Welfare and Ethics Committee of the Japan Racing Association Equine Research Institute (20–10).

### Horses and transport

Six Thoroughbred geldings of median age 5.5 years (range 5–7 years) with median body weight 534 kg (range 459–640 kg) before transport were included in the current study. All horses were from the Japan Racing Association’s Equine Research Institute herd, resident in the same location for more than 12 months, and had prior experience of transportation (i.e., ≥3 journeys/horse). Each horse was judged free of clinical disease, based on a clinical examination performed by two veterinarians, and hematology and serum biochemistry results within normal reference ranges. Except during transportation, horses were housed in individual stalls (2.7 × 3.6 m) with straw bedding and kept in a 17 × 22 m yard for approximately 6 h each day. All horses received 0.5 kg oats, 1 kg of pelleted feed, and 3 kg of timothy hay in the morning (8:00 AM) and evening (4:00 PM). Water was available *ad libitum*.

**Figure 1:**
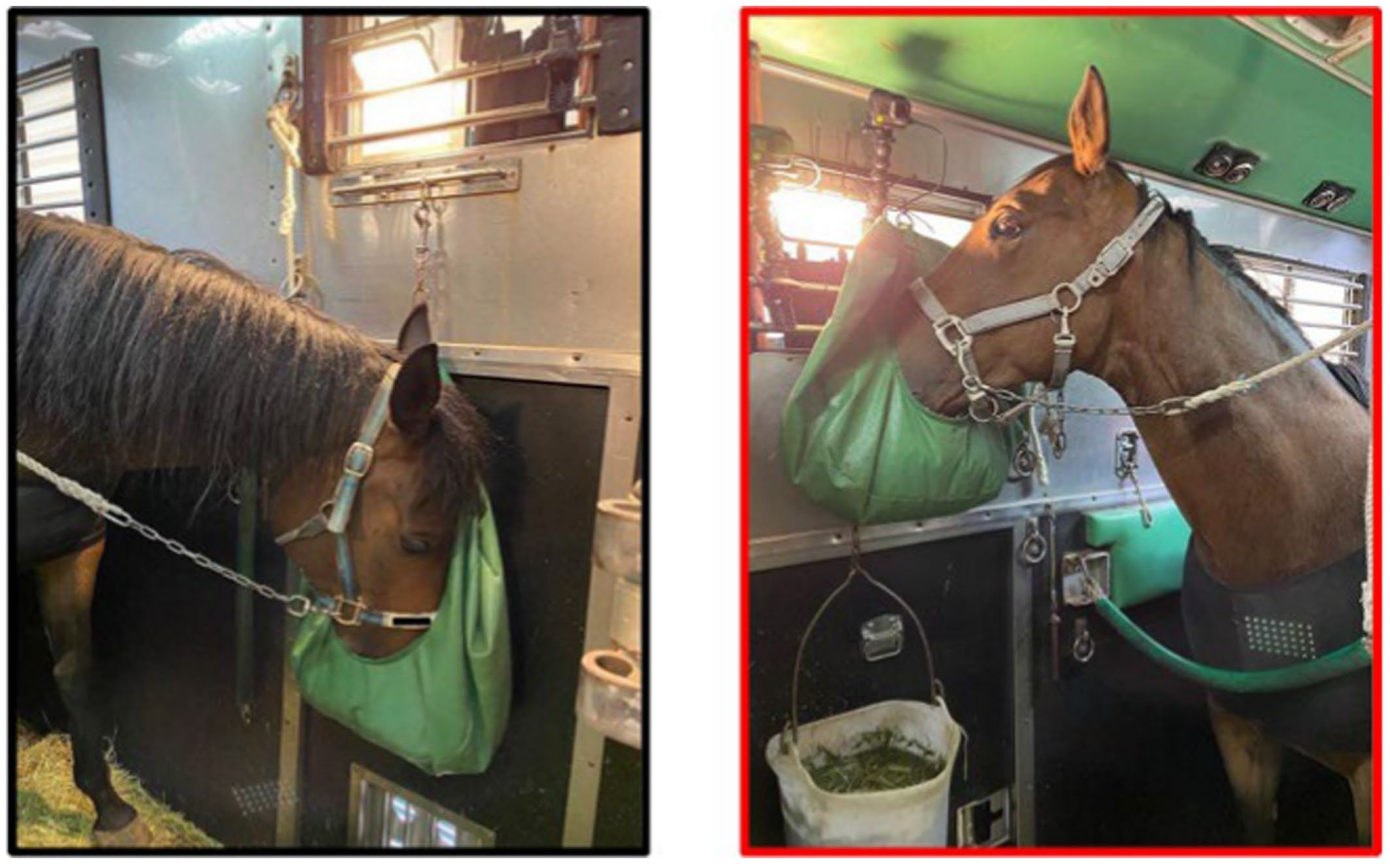
Photos of each treatment (left; long-rope, right; short-rope). In the long-rope group, the length of the rope was 95 cm, allowing the horses to lower their heads to the level of their carpal joints, while the length of the rope was 50 cm in the short-rope group, which prevented the horses lowering their heads below wither height. Hay was positioned at a height of 0.9 m above the floor for the long-rope group, and at 1.6 m for the short-rope group so that all horses were able to eat freely during the transport. In both groups, behaviors were recorded by a camera set on the front side of the horse at the height of 1.7 m from the floor.

In a randomized crossover study, horses were transported twice, with the 4-week separation between trips considered sufficient to ensure recovery from the first transport event. Both transport events covered the same round-trip of approximately 1,400 km, departing from the Equine Research Institute at 3:00 PM and returning to the same location after 22 h. During the first and last 4 h of the journey, horses were monitored using a camera (GoPro HERO 7 Black, GoPro Inc., SM, California, United States) mounted on the wall of the truck directly in front of each horse at the height of 1.7 m from the floor. Each subject was monitored by one camera as described below. Horses were transported in a commercial van that had six horse stalls (0.9 × 2.3 m), and hay was hung over the wall so that horses could eat freely during transport. Horses faced forward and were cross-tied by lead ropes attached to the horse’s head collar (Figure 1). By adjusting the length of the ropes, three horses were randomly allocated by ballot into a “short-rope” group (the length of each rope was 50 cm), which prevented the horses lowering their heads below wither height. A bag containing hay and 0.5 kg pelleted feed was positioned at a height of 1.6 m above the floor for this group. The remaining three horses comprised the “long-rope” group (the length of each rope was 95 cm), allowing the horses to lower their heads such that their nose was level with their carpus (Figure 1). The feed bag was positioned at 0.90 m height for this group. Using a bucket, water was offered to horses by a groom (5 min for each horse) every 4 h during a mandated 30-min rest for drivers. Hay and pelleted food were added to bags during these rests so that horses could eat hay or pelleted feed throughout the journey. Horses were not unloaded during driver rest stops, and the length of the rope did not change during the rest. Horses were allocated to the reciprocal treatment group for the second transport event. In addition, temperature and relative humidity inside the truck were measured during these rests by a portable monitoring device (WBGT-213B, Kyoto Electronics Manufacturing, Kyoto, Japan).

Body weight was measured by electronic scales and rectal temperature was recorded by digital thermometer. Both parameters were recorded for each horse immediately before departure and upon arrival.

### Behavioral parameters

Video recordings were analyzed by one researcher (Y.T.) using a time window defined as the first 15 min of each hour of recorded footage; that is, four 15-min epochs from the first 4 h of the journey, and four 15-min epochs from the final 4 h of the journey (a total of 120 min). The behaviors were analyzed using the ethogram reported in Table 1 (27). Behavioral parameters including duration of head up (the head was above the withers) and down (the head was below the withers), eating hay with head down or with the head up, and the frequency of licking episodes (defined as opening mouth with extension and retraction of tongue without eating anything) were measured. The summation of data from each 15-min epoch for each behavioral parameter was used for statistical analysis.

**Table 1:** Behavior sampling ethogram.

Behavior	Description
Licking	Opening of mouth with extension and retraction of tongue, lip smacking without tongue extension, lateral jaw movements involving partial opening of the lips (27)
Head down duration	Horses stay their head at the level or below withers height (20)
Head up duration	Horses stay their head above withers height
Eating with head down	Mediolateral or vertical movement of jaw (36) soon after grabbing hay with their head at the level or below withers height
Eating with head up	Mediolateral or vertical movement of jaw (36) soon after grabbing hay with their head above withers height

Licking was analyzed as a behavioral event and expressed as frequency (n/15 min), while other behavioral states were expressed as duration (min/15 min). All parameters were monitored 15 min every 1 h from the first 4 h of the journey and the final 4 h of the journey. They have been summed up for statistical analysis (events: total n/120 min, and duration total min/120 min).

### Bacteriology

In order to assess the effect of head restraint on respiratory tract contamination, tracheal washes (TW) were collected 7 days before transportation and immediately on return from transportation. Horses were physically restrained in a stock with a nose twitch, and a TW was obtained by lavage and aspiration of 50 mL of room temperature 0.9% saline instilled into the distal trachea via an extension tube with stopcock (TS-WR2527, Terumo, Tokyo, Japan) passed through the instrument channel of a 3 m endoscope (VQ-930c, Olympus, Tokyo, Japan). Because this treatment could not be conducted on all horses simultaneously, horses were tied to the stall wall immediately after unloading, to prevent lowering of their head until the TW collection was completed. The endoscope and the instrument channel were cleaned with 70% alcohol and tap water between examination of each horse.

Each TW sample was treated for 15 min with a 3-fold volume of semi-alkaline protease solution (Suputazyme, Kyokuto Pharmaceutical Industries Co. Ltd.) to dissolve mucus. Ten-fold serial dilutions of the treated TW were made with sterile saline, and 100 μL of each dilution was plated on two sets of Colombia agar plates with 5% horse blood. One set was incubated in 5% CO_2_ in air and another set was incubated anaerobically for 24 h at 37°C. The number of colony-forming-units (CFU) per mL (converted to log_10_ CFU/ml) was determined by counting the total number of bacterial colonies on the plates. Further, different organisms, recognized by discrete colony types, were subcultured and identified by MALDI-TOF MS (MALDI-Biotyper, Bruker, MA, United States). Organisms that could not be identified with MALDI-TOF MS were characterized with Gram staining, air condition for growth, and microscopic morphology.

### DNA extraction

Total bacterial DNA was extracted using HostZERO Microbial DNA kit (Zymo Research, Irvine, CA, United States) in accordance with manufacturer’s instructions from concentrated TW samples prepared as follows. Four mL of TW sample was centrifuged at 20,000 x g for 3 min, before removal of 3.8 mL supernatant. The pellet was resuspended in the remaining 200 μL of supernatant, and the diluent was used as the concentrated TW sample. The extracted DNA was stored at −20°C until amplification and sequencing.

### Amplification

Metagenomic analysis was based on amplification of 16S rRNA gene from the extracted DNA. The amplification step included two PCRs. For the first PCR, 50 μL of reaction mixture included 2 μL of the extract DNA, 200 μM of forward primer 27f (5′-AGAGTTTGATCCTGGCTCAG-3′) (28), 200 μM reverse primer 1492r (5′-AGAGTTTGATCCTGGCTCAG-3′) (28), 25 μL of PrimSter^®^ HS(Premix) (Takara Bio Inc., Shiga, Japan). The first PCR was performed using a 2 m initial denaturation at 94°C followed by 20 cycles of 94°C for 30 s, 50°C for 30 s, and 72°C for 90 s, with a final extension of 5 m at 72°C. Amplicons were purified with Agencourt AMPure XP beads (Beckman Coulter Inc., ON, Canada). The second PCR was based on a fusion PCR method described by Thermo Fisher Scientific^1^ that combined the A adapter, trP1 adapter, and barcode sequences to amplicons for sequencing. The PCR used a combination of 12 barcoded primers with the A adapter and a reverse primer with the trP1 adapter. Sequences of the primers are listed in Supplementary Table S1. Primers were based on a previous report (29), and amplicons included V4 and V5 regions of the 16S rRNA gene. For the second PCR, 50 μL of reaction mixture included 1 μL of the purified amplicon solution, 200 μM of forward primer U515F_A_BCx_Fw (where *x* corresponds numbering of barcode listed in Supplementary Table S1), 200 μM reverse primer U909_trP1_Rv, 25 μL of PrimSter^®^ HS(Premix). The second PCR was performed under the following conditions: a 2 min initial denaturation at 94°C followed by 20 cycles of 94°C for 15 s, 56°C for 5 s, and 72°C for 60 s, with a final extension of 2 m at 72°C. The second PCR amplicons were purified with Agencourt AMPure XP beads (Beckman Coulter Inc.) twice and then stored at –20°C until use.

### Metagenome analysis

The purified second PCR amplicons were sequenced by Ion PGM (Thermo Fisher Scientific K.K., Tokyo, Japan) with 400-bp chemistry using an Ion PGM Hi-Q View Sequencing Kit (Thermo Fisher Scientific K.K.) with on 316 Chip Kit v2 BC (Thermo Fisher Scientific K.K.), in accordance with manufacturer’s instructions. Quality checking and homology analysis with the Basic Local Alignment Search Tool (BLAST algorithm) for bacterial 16S rRNA from the obtained FASTAQ sequencing files were performed using Homology analysis tool (World Fusion Co. LTD., Tokyo, Japan). Relative abundance and *α*-diversity based on Simpson’s index and Shannon index were calculated using Metagenome @KIN software (World Fusion Co. LTD.).

### Cortisol concentration determination

Blood was collected by jugular venipuncture just before departure and immediately after arrival. Plasma cortisol concentration was measured by the Laboratory of Racing Chemistry (Tochigi, Japan) using a high-speed liquid chromatograph-tandem mass analysis system (Nexera X2, Shimadzu, Kyoto, Japan, and QTRAP4500, SCIEX, Framingham, MA, United States).

### Statistical analysis

The effects of transport condition (long-rope or short-rope), time (pre transport vs. post transport) and their interaction on body weight, bacterial numbers in TW (log_10_ CFU/ml), Simpson index, Shannon index, and cortisol concentration were evaluated by mixed model (PROC MIXED). These were treated as fixed effects, while each horse was considered as a random effect. When the interaction was significant, Tukey’s multiple comparisons were performed as *post hoc* tests. We confirmed that residuals of these parameters were distributed normally by Shapiro–Wilk tests (*p* > 0.06). Behavioral data were tested for normal distribution using Shapiro–Wilk test (*p* > 0.11). In order to see the effect of the transport condition on eating behavior, a paired *t*-test was used; total time eating was compared between groups. Wilcoxon signed-rank test was used to compare the frequency of total licking behaviors monitored (n/120 min) in each group. All statistical analyses were conducted by commercial software (SAS 9.4, SAS Institute Inc., Cary, NC, United States) and a *p-*value of <0.05 was considered significant. Mean ± standard deviation was used to report data distributed normally, and median (IQR, and range) was used to report non-parametric data.

## Results

Temperature and relative humidity inside the truck ranged between 18.0–29.4°C and 46–73% in the first journey, respectively, and between 23.2–26.5°C and 54–74% in the second journey. No horses were injured during transportation. A significant (*p* < 0.001) reduction in body weight was observed after transportation in both groups (long-rope; 25.0 ± 6.7 kg, short-rope; 26.0 ± 4.3 kg), but there was no effect of transport condition (*p* = 0.91) or interaction (*p* = 0.72, Supplementary Figure S1). Two horses were coughing frequently at unloading (Horse 1 and 4 in Supplementary Table S2), and one horse had a rectal temperature of 38.5°C (Horse 1 in Supplementary Table S2). Both horses were in the short-rope group and recovered the next day without treatment. No horses in the long-rope group showed any clinical signs of respiratory disorders at unloading.

### Behavioral parameters

Horses in the short-rope group spent all time with their heads up (120 min), whereas horses in the long-rope group spent a mean of 59.8 ± 15.4 min with their heads up and 60.2 ± 15.5 min with their heads down. This indicates that they spent approximately 50% of the monitoring time with their heads lowered, below the level of their withers. Total time spent eating hay did not differ between groups (long-rope: 60.3 ± 9.66 min vs. short-rope: 48.4 ± 10.1 min, *p* = 0.09). In the long-rope group, time spent eating hay with the head down was 47.5 ± 11.1 min, while that of eating hay with the head up was 12.8 ± 6.67 min. Horses in the short-rope group could not eat with their heads down. No horse consumed a measurable amount of water during the rest stops on either trip. The licking frequency in the short-rope group (median;139, range; 80–525) was significantly higher than in the long-rope group (median; 62, range; 12–122, *p* = 0.01).

### Quantitative bacteriology of TW samples

**Figure 2:**
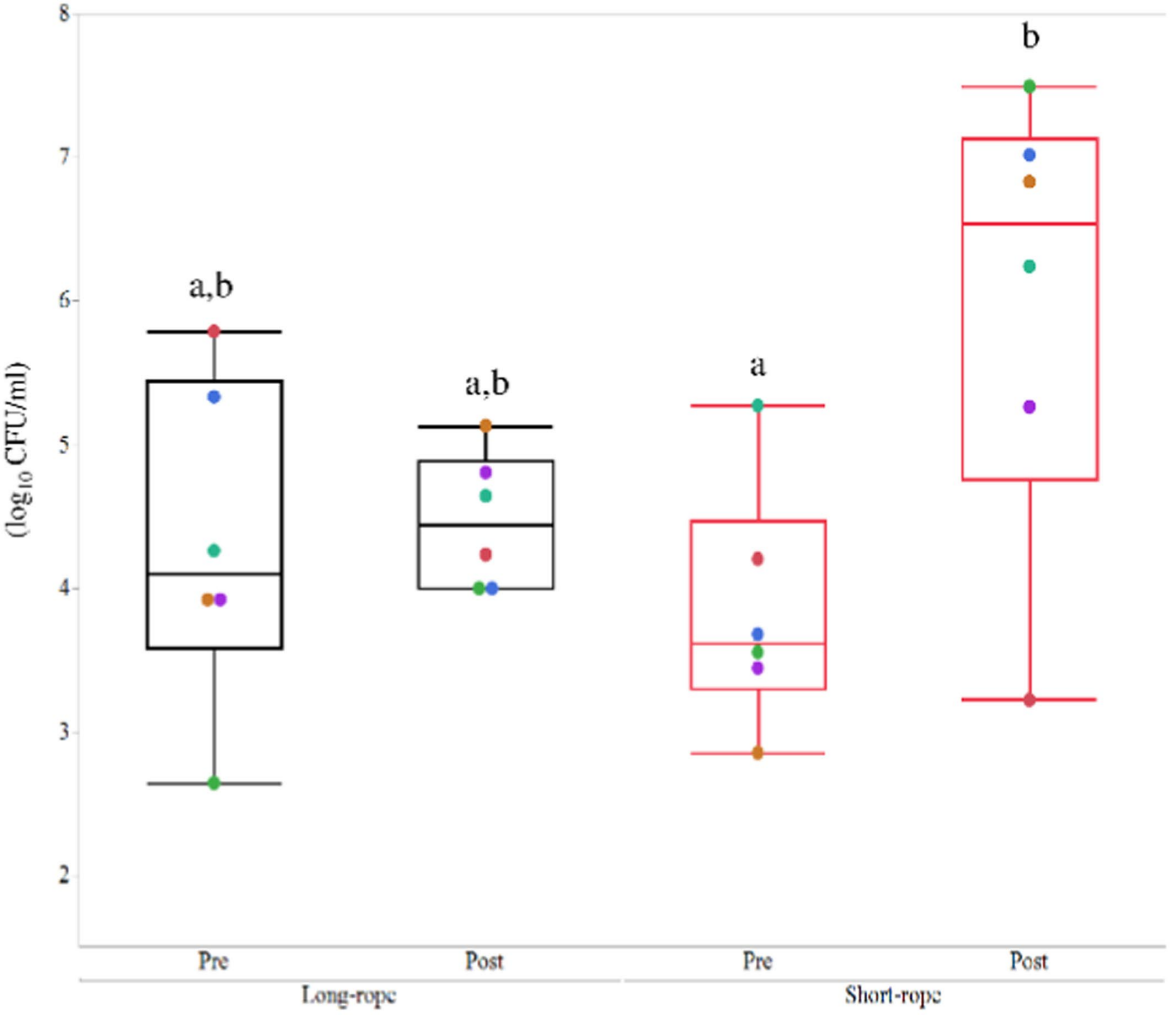
Mean ± standard deviation of changes of colony forming unit (CFU) in the long-rope group and short-rope group. Different colored marks data of individual horses. Different letters indicate significant difference (*p* < 0.05).

All TW samples before transport were clear and transparent, whereas those obtained following transportation were turbid and, in the short-rope group, yellow in color (Supplementary Figure S2). The effect of time on bacterial numbers in TW samples was significant (*p* = 0.01) and a significant interaction between time and transport condition was observed (*p* = 0.04). *Post hoc* analysis revealed that TW samples from post-transport in the short-rope group included more bacteria than pre-transport in the short-rope group (*p* = 0.02) (Figure 2). Total bacterial numbers in tracheal wash samples from the short-rope group increased by more than 100-fold after transportation (Pre: 3.84 ± 0.83 log_10_ CFU/ml, Post: 6.01 ± 1.57 log_10_ CFU/ml), whereas no increase was observed in the long-rope group after 22 h of transport (Pre: 4.31 ± 1.12 log_10_ CFU/ml, Post: 4.47 ± 0.46 log_10_ CFU/ml). Bacterial species identified in TW samples varied for each individual horse, but the most commonly identified isolates were *Streptococcus* spp. and members of family *Pasteurellaceae* (Supplementary Table S2).

### Bacteriology microbiome

**Figure 3:**
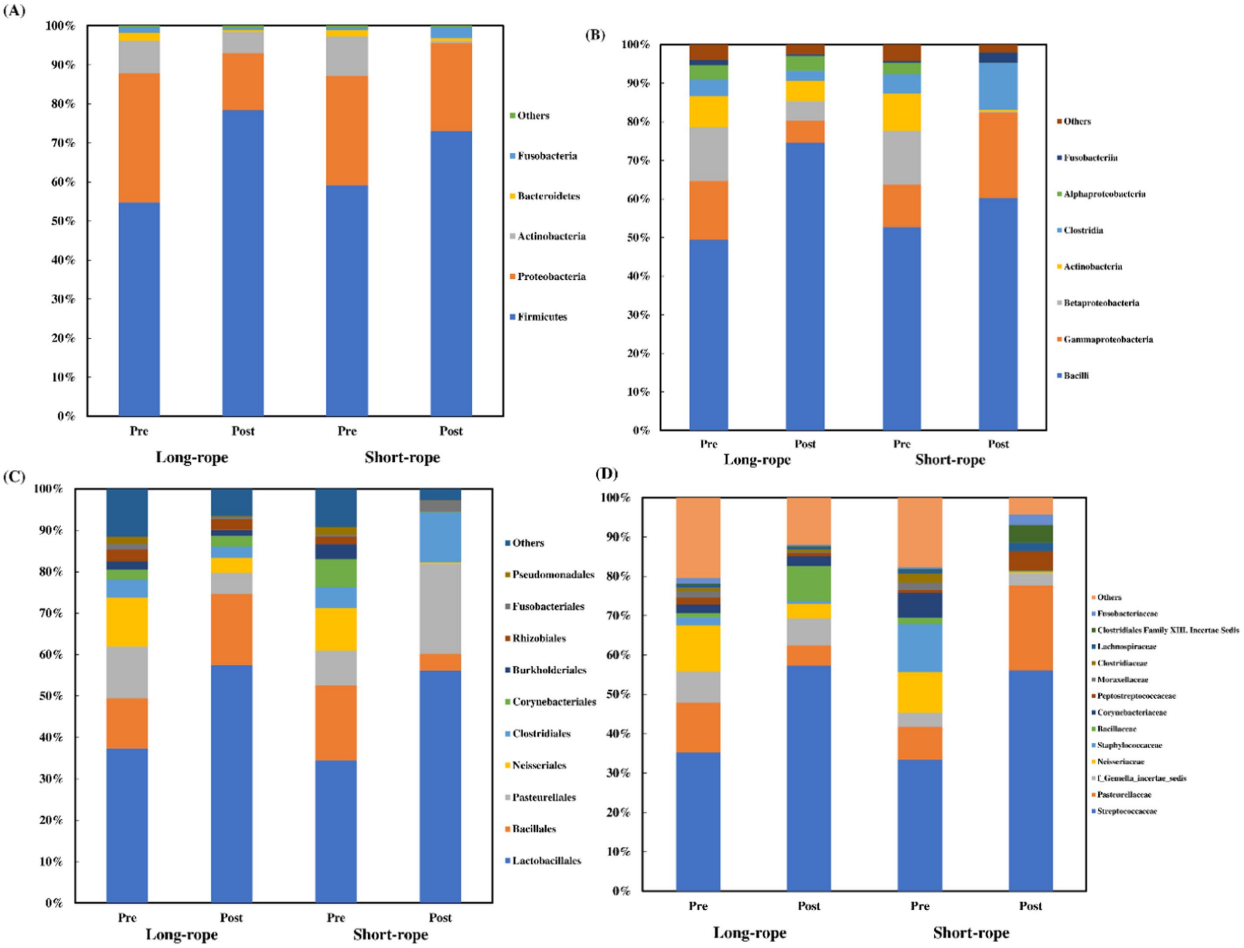
Changes of mean relative abundance of predominant phyla **(A)**, classes **(B)**, orders **(C)**, and families **(D)** in tracheal wash in each group. Standard deviations were omitted for clarity. Others: bacterial taxa with <1.5% abundance.

**Figure 4:**
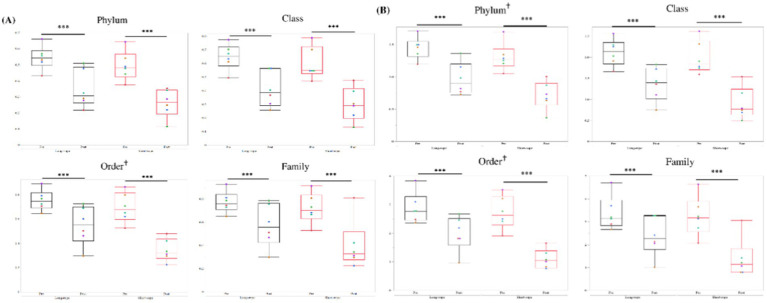
Changes in *α*-diversity (**A**; Simpson index, **B**; Shannon index) in the long-rope group and short-rope group. *** indicates significant changes by transport (*p* < 0.001). † at the right shoulder of order indicates significant differences by transport condition (*p* < 0.05). No interactions between transport and transport condition were observed in any levels. Different color dots show different horses.

A median of 82,981 sequences was obtained in each TW sample (min: 37,896; max: 175,040). Five phyla, 7 classes, 10 orders, and 13 families, had a mean relative abundance of over 1.5% (Figure 3). Three phyla (Firmicutes, Proteobacteria, and Actinobacteria) represented over 95% of the total abundance, and Firmicutes was the dominant phylum after transport in both groups (Figure 3A). Bacilli (Figure 3B), Lactobacillales (Figure 3C), and *Streptococcaceae* (Figure 3D) were dominant at class, order and family level, respectively, regardless of transport condition. In both groups, except for class level, the dominant member before transport showed the largest increase in relative abundance in each level (Firmicutes; +24% in long-rope and +14% in short-rope, Bacilli; +20% in long-rope and +22% in short-rope, *Streptococcaceae;* +22% in long-rope and +23% in short-rope, respectively). At class level, the largest increase was observed in Lactobacillales in the long-rope (+25%), while Gammaproteobacteria showed the largest increase in the short-rope group (+11%). Figure 4 shows the effect of time on Simpson index and Shannon index at phylum, class, order and family levels (all; *p* < 0.01). Regarding the effect of transport condition, Simpson index at order level (*p* = 0.01), Shannon index at phylum (*p* = 0.03) and order level (*p* = 0.02) were significantly lower in the short-rope group than the long-rope group, while no significant differences due to restraint with long-or short-rope were observed (*p* > 0.08). There were no significant interactions at any levels either Simpson index or Shannon index (Figure 4, *p* > 0.15).

### Cortisol response

**Figure 5:**
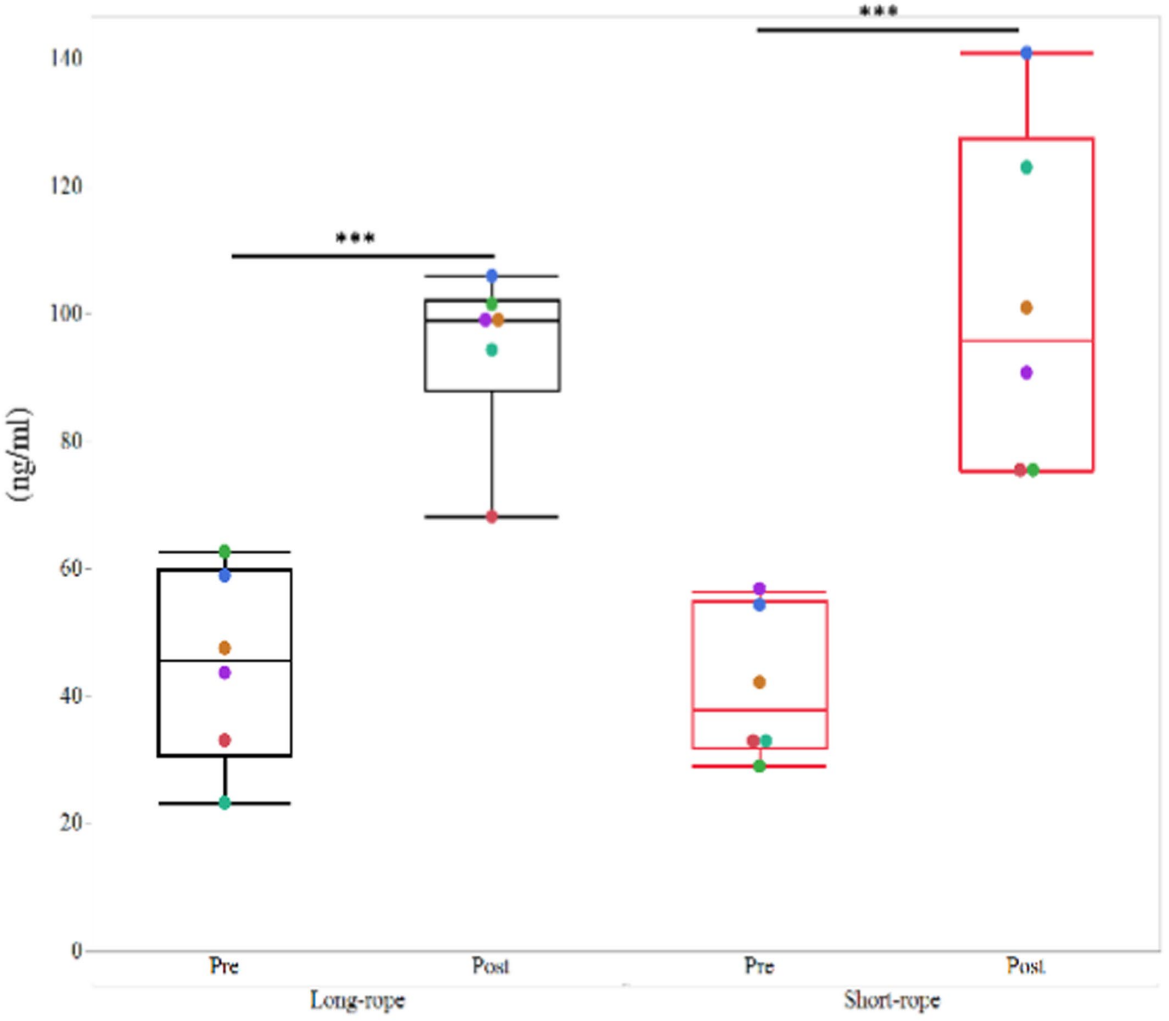
Plasma cortisol concentrations in the long-rope group and short-rope group. *** indicates significant changes by transport (*p* < 0.001). Different color dots show different horses.

Cortisol concentrations were increased (*p* < 0.001) after transportation, but no significant effect of transport condition (*p* = 0.84) or their interaction (*p* = 0.49) was observed (Figure 5).

## Discussion

In the present study, horses in the short-rope group spent all the journey with their head in an elevated position and two horses showed clinical signs of respiratory disorders at unloading. Clinical signs of respiratory tract compromise were not observed in horses restrained with a longer rope. Further, horses in the long-rope group demonstrated less stress-related behavior and greater numbers of bacteria in tracheal wash samples after transportation. These findings demonstrate that loose head restraint during transportation ameliorated the accumulation of airway secretions and associated increased bacterial load observed after transportation in short-rope group horses, and this strategy is likely to protect horses from transport-associated respiratory disease.

Transport significantly reduced the diversity of microbiota in the trachea, which is consistent with previous reports (20, 30). As there are a vast number of commensals and potential pathogens that inhabit the mucosa of the respiratory tract, a delicate equilibrium has to be maintained between immune sensing and tolerance of non-pathogenic commensals with being specific host-microbiota interactions (31). The decreased diversity, which can be considered to be disrupted fine balance between microbiota, is consistent with the observed culture results, and reflects preferential multiplication of specific bacteria, including members of *Streptococcaceae* and *Pasteurellaceae*, well recognized as the most common pathogens for transport-related respiratory disease (22, 24). Although the diversity of microbiota in the trachea did not differ between the two groups, the total number of bacteria was significantly increased in TW samples from short-rope horses. With head and neck restrained at a high position, tracheal mucociliary clearance mechanisms could be impaired, as has been demonstrated by a previous study (19).

Because lower head posture (cranial trachea lower than the caudal trachea) greatly accelerates mucociliary clearance (19), low head position would be expected to improve tracheal hygiene. However, intermittent lowering of the head for 30 min every 6 h did not prevent multiplication of organisms in trachea (18). In the current study, horses in the long-rope group spent almost 50% of the monitoring time in a head down position, including a greater length of time eating with their heads down than with heads in an elevated position. Horses restrained with a short-rope were unable to lower their heads, suggesting that the inability to adopt this postural adaptation was sufficient to facilitate prevent clearance of airway secretions.

In addition to permitting a head-down posture, we further speculate that freedom of head and neck movement may mitigate transport-stress. Increased licking frequency was observed in the short-rope group, relative to behaviors demonstrated by horses in the long-rope group. Licking has been validated as a stress-related behavior (27), and a good indicator of stress during transport (20). According to a previous report, licking frequency was negatively correlated with total duration of head lowering during 8 h transport (20), and may suggest that horses with tight head restraint feel stressed or frustrated due to the restriction of head movement during transport. Although blood cortisol level is a reliable marker of stress in animals, we could not detect a difference between short-and long-rope restraint in the current study. We speculated this is because we compared licking behavior frequency during the journeys, while cortisol data only before and after the journey. When horses feel stressed in a novel environment, such as loading for transportation, the hypothalamus-pituitary–adrenal axis is activated, leading to the release of adrenocorticotrophic hormone and cortisol (32). During transport, increased blood cortisol concentrations are observed 1–3 h after departure (11, 26, 33) and cortisol response gradually decreases with prolonged exposure (11). Therefore, determination of cortisol response during travel and comparison of the area under the curve of serum or salivary cortisol may have better sensitivity to detect overall stress levels during transport, as has been previously reported for salivary cortisol concentrations (5). Additionally, cortisol does not always reflect stress level. Padalino et al. demonstrated that stress-related behaviors including licking correlated with decreased gastrointestinal sounds after transport, but not with serum cortisol (25), suggesting that behavioral changes might be more sensitive indicators of stress than endocrine response. There may be cases where observing behavioral changes is more appropriate for detecting certain types of stress.

Although there are limited data on normal equine airway microbiota (21, 30, 34, 35), the findings of the current study were different to expectations, even prior to transportation. Whereas previous studies have reported phylum Proteobacteria (21, 30) and family *Moraxellaceae* or *Pasteurellaceae* (20, 30) are the most dominant in healthy horses, phylum Firmicutes and family *Streptococcaceae* were dominant before and after transport in our horses. This observation suggests that airway microbiota might be affected by stable environment, as has been previously suggested (34). Although all horses in the current study were stabled in the same location and fed in the same way for more than 12 months, large variations were still observed in results obtained from individual animals.

For the short-rope group, the hay net was always close to each horse’s nose, while horses in the long-rope group were able to move their nose further from the hay. Although concern has been expressed that feed within the respirable zone might increase the bacterial load within the horse’s airways, our findings do not support this concern because the increased bacterial numbers observed in TW samples were due to increased numbers of oropharyngeal organisms, rather than increased environmental organisms, as has been previously reported (14, 18–20). Nonetheless, the potential risk exists that, due to increased numbers of environmental organisms or increased dust and respirable debris, there may be an increased burden on tracheal clearance mechanisms associated with proximity to hay (short-rope group), and this might exacerbate postural effects on tracheal mucociliary clearance. Despite differences in time spent eating with the head in lowered position between groups, there was no difference observed between groups associated with time spent eating, nor with body weight lost during transportation. Horses in both groups were eating for approximately 50% of the observational windows, but both groups lost approximately 5% body weight during the trip, an observation that is likely related to their refusal to drink water offered during the journey.

Our findings should be interpreted with due caution given study limitations including a small sample size of horses. More research covering other breeds, mares and stallions, an increased age range and horses with diverse travel experience, would be needed to apply our results to the broad equine population. Secondly, recording of behavioral parameters throughout the full duration of transport was impossible because of recording memory shortage. This limitation precluded evaluation of effects of transport time on behavioral parameters, as has been previously reported (20). However, based on changes observed in this previous study (20), our focused evaluation of behavior at the beginning and end of the journey is likely to have provided sufficient opportunity to document stress related behaviors during early and final stages of transport and is therefore likely representative of the entire journey (11, 20). Thirdly, although there was no difference between groups in time spent eating or body weight loss during the trip, the total amount of hay, concentrate, and water consumed by horses during the study was not recorded. Forth, we did not evaluate other possible parameters that may be changed by transport, such as microbiome *β* diversity (21, 30) or other stress related behaviors including touching the head rope (20). In particular, analysis of β diversity would help us quantitatively understand how the composition of microbial communities was altered in response to long-or short-rope restraint. Our findings, however, were entirely comparable with comparable transportation studies (20). Finally, cytology was not performed on TW samples in the current study, although this has varied little in similar studies on confined (17, 18) and transported (13, 23) horses. To date, cytology changes have not indicated increased inhalation of environmental bacteria or respirable debris.

Tight head restraint and elevated head position were associated with increased bacterial numbers in TW samples in this study. Our findings suggest that loose head restraint and feeding of horses from a height to encourage lowering of the head during transportation is likely to ameliorate the well-documented increase in tracheal bacterial load associated with transportation, and to reduce stress behaviors associated with head restraint during the long transport.

## Data Availability

The original contributions presented in the study are included in the article/Supplementary material, further inquiries can be directed to the corresponding author.

## Footnotes

^1^https://ioncommunity.thermofisher.com/docs/DOC-1844
