# Supplementary material for: Increased freedom of head movement mitigates stress and bacterial load in the airways of horses during transport
Source: Front Vet Sci. 2024 Oct 4;11:1477653. doi: 10.3389/fvets.2024.1477653 (PMC11486923; doi:10.3389/fvets.2024.1477653)
Supplement: Supplementary file 1 [file Data_Sheet_1.docx]

Supplementary Material

# Supplementary Tables

**Table S1.** Primers used for a fusion PCR in this study.

| Primers | Sequences |
| --- | --- |
| U515F_A_BC1_Fw | CCATCTCATCCCTGCGTGTCTCCGACTCAGCTAAGGTAACGATGTGYCAGCMGCCGCGGTA |
| U515F-2_A_BC2_Fw | CCATCTCATCCCTGCGTGTCTCCGACTCAGTAAGGAGAACGATGTGYCAGCMGCCGCGA |
| U515F-3_A_BC3_Fw | CCATCTCATCCCTGCGTGTCTCCGACTCAGAAGAGGATTCGATGTGYCAGCMGCCGCGGTA |
| U515F-4_A_BC4_Fw | CCATCTCATCCCTGCGTGTCTCCGACTCAGTACCAAGATCGATGTGYCAGCMGCCGCGGTA |
| U515F-5_A_BC5_Fw | CCATCTCATCCCTGCGTGTCTCCGACTCAGCAGAAGGAACGATGTGYCAGCMGCCGCGGA |
| U515F-6_A_BC6_Fw | CCATCTCATCCCTGCGTGTCTCCGACTCAGCTGCAAGTTCGATGTGYCAGCMGCCGCGGTA |
| U515F-7_A_BC7_Fw | CCATCTCATCCCTGCGTGTCTCCGACTCAGTTCGTGATTCGATGTGYCAGCMGCCGCGGTA |
| U515F-8_A_BC8_Fw | CCATCTCATCCCTGCGTGTCTCCGACTCAGTTCCGATAACGATGTGYCAGCMGCCGCGGTA |
| U515F-9_A_BC8_Fw | CCATCTCATCCCTGCGTGTCTCCGACTCAGTGAGCGGAACGATGTGYCAGCMGCCGCGGTA |
| U515F-10_A_BC10_Fw | CCATCTCATCCCTGCGTGTCTCCGACTCAGCTGACCGAACGATGTGYCAGCMGCCGCGGTA |
| U515F-11_A_BC11_Fw | CCATCTCATCCCTGCGTGTCTCCGACTCAGTCCTCGAATCGATGTGYCAGCMGCCGCGGTA |
| U515F-12_A_BC12_Fw | CCATCTCATCCCTGCGTGTCTCCGACTCAGTAGGTGGTTCGATGTGYCAGCMGCCGCGGTA |
| U909R_trP1_Rv | CCTCTCTATGGGCAGTCGGTGATCCCCGYCAATTCMTTTRAGT |

Underlines indicate barcode sequence of the primers. For forward primer, U515_A_BCx_Fw, where x corresponds numbering of barcode were used. For reverse primer, U909_trP1_Rv was used.

**Table S2.** Colony forming units (CFU) of bacterial species in tracheal wash, identified on blood agar medium before and after transport in each horse.

|  | Long-rope | | | | Short-rope | | | |
| --- | --- | --- | --- | --- | --- | --- | --- | --- |
|  | Pre | | Post | | Pre | | Post | |
| Horse | Bacteria | CFU/ml | Bacteria | CFU/ml | Bacteria | CFU/ml | Bacteria | CFU/ml |
| Horse 1 | Streptococcus sp. | 400 | Bacillus sp. | 4400 | Streptococcus sp. | 1600 | Actinobacillus equuli | 22400000 |
|  | Streptococcus luteriensis | 40 | Streptococcus sp. | 3200 | Streptococccus criceti | 1200 | Streptococcus sp. | 7400000 |
|  |  |  | Staphylococcus xylosus | 2400 | Facultative Gram positive rod | 800 | Facultative Gram positive rod | 800000 |
|  |  |  |  |  |  |  | Actinobacillus arthritidis | 640000 |
| Horse 2 | Aerobic Gram negative coccoid | 2400 | Streptococccus sp. | 36000 | Streptococcus sp. | 2000 | Streptococcus sp. | 84000 |
|  | Streptococcus equinus | 2000 | Actinobacillus suis | 20000 | Streptococcus luteriensis | 800 | Escherichia coli | 28000 |
|  | Streptococcus sp. | 1200 | Actinobacillus sp. | 4000 |  |  | Actinobacillus equuli | 24000 |
|  | Staphylococcus xylosus | 800 | Actinobacillus genomespeies | 4000 |  |  | Facultative Gram positive coccoid | 24000 |
|  | Pasteurella caballi | 800 |  |  |  |  | Streptococcus orisasini | 16000 |
|  | Escherichia coli | 800 |  |  |  |  | Streptococcus criceti | 8000 |
| Horse 3 | Streptococcus sp. | 460000 | Streptococcus sp. | 5600 | Streptococcus sp. | 12000 | Anaerobe Gram positive coccoid | 680 |
|  | Facultative Gram positive coccoid | 116000 | Streptococcus orisasini | 4400 | Facultative Gram positive coccoid | 2000 | Facultative Gram negative rod | 480 |
|  | Streptococcus criceti | 40000 | Facultative Gram negative positive rod | 4000 | Streptococcus orisasini | 1600 | Streptococcus sp. | 440 |
|  |  |  | Pasteurella caballi | 1200 | Actinobacillus equuli | 400 | Streptococcus refensis | 80 |
|  |  |  | Streptococcus salivarius | 1200 |  |  |  |  |
|  |  |  | Actinobacillus rossii | 400 |  |  |  |  |
|  |  |  | Streptococcus zooepidemicus | 400 |  |  |  |  |
| Horse 4 | Streptococcus salivarius | 108000 | Pasteuerlla cabali | 6000 | Staphylococcus xylosus | 2000 | Streptococcus sp. | 8000000 |
|  | Streptococcus sp. | 56000 | Streptococcus sp. | 3200 | Staphylococcus sp. | 1600 | Actinobacillus equuli | 1600000 |
|  | Facultative Gram positive coccoid | 36000 | Actinobacillus genomespecies | 800 | Streptococcus equinus | 800 | Pasteurella caballi | 800000 |
|  | Pasteurella caballi | 16000 |  |  | Streptococcus salivarius | 400 |  |  |
|  |  |  |  |  |  |  |  |  |
| Horse 5 | Facultative Gram positive coccoid | 4800 | Pasteurella caballi | 28000 | Facultive Gram positive coccoid | 72000 | Nicoletella semolina | 1600000 |
|  | Pasrteurella caballi | 4800 | Streptococcus sp. | 12000 | Streptococcus sp. | 68000 | Streptococcus sp. | 92000 |
|  | Streptococcus salivarius | 3600 | Veillonella sp. | 4000 | Pasteurella caballi | 32000 | Actinobacillus equuli | 28000 |
|  | Facultative Gram positive rod | 2400 |  |  | Veillonella pervula | 16000 | Facultative Gram positive coccoid | 24000 |
|  | Staphylococcus sp. | 1600 |  |  |  |  | Streptocoocus zooepidemicus | 640 |
|  | Streptococcus luteriensis | 800 |  |  |  |  |  |  |
|  | Streptococcus zooepidemicus | 320 |  |  |  |  |  |  |
| Horse 6 | Pasteurella caballi | 2800 | Streptococcus sp. | 92000 | Streptococcus sp. | 640 | Streptococcus sp. | 6760000 |
|  | Streptococcus sp. | 2400 | Actinobacillus equuli | 32000 | Streptococcus equorum | 40 |  |  |
|  | Actinobacillus rossi | 1600 | Pasteurella caballi | 12000 | Bacillus cereus | 40 |  |  |
|  | Facutative Gram negative rod | 1200 |  |  |  |  |  |  |
|  | Actinobacillus equuli | 800 |  |  |  |  |  |  |

# Note that Horse 1 and 4 were coughing frequently at unloading and Horse 1 had a rectal temperature of 38.5℃ after transport in short-rope condition.

# Supplementary Figures


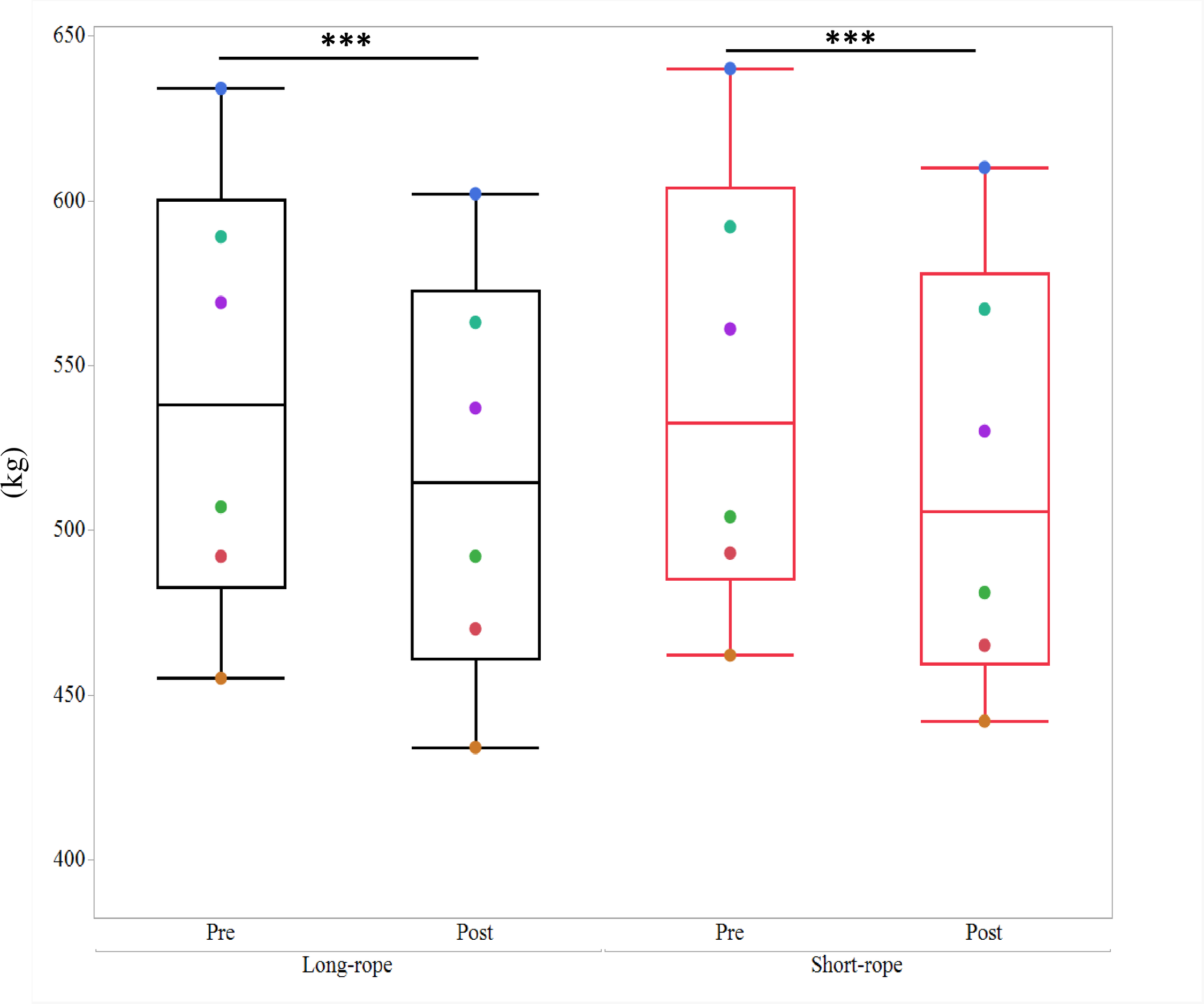


**Figure S1.** Mean ± standard deviation of body weight in the long-rope and short-rope group. *** indicates significant changes by transport (P < 0.001). Different color dots show different horses.


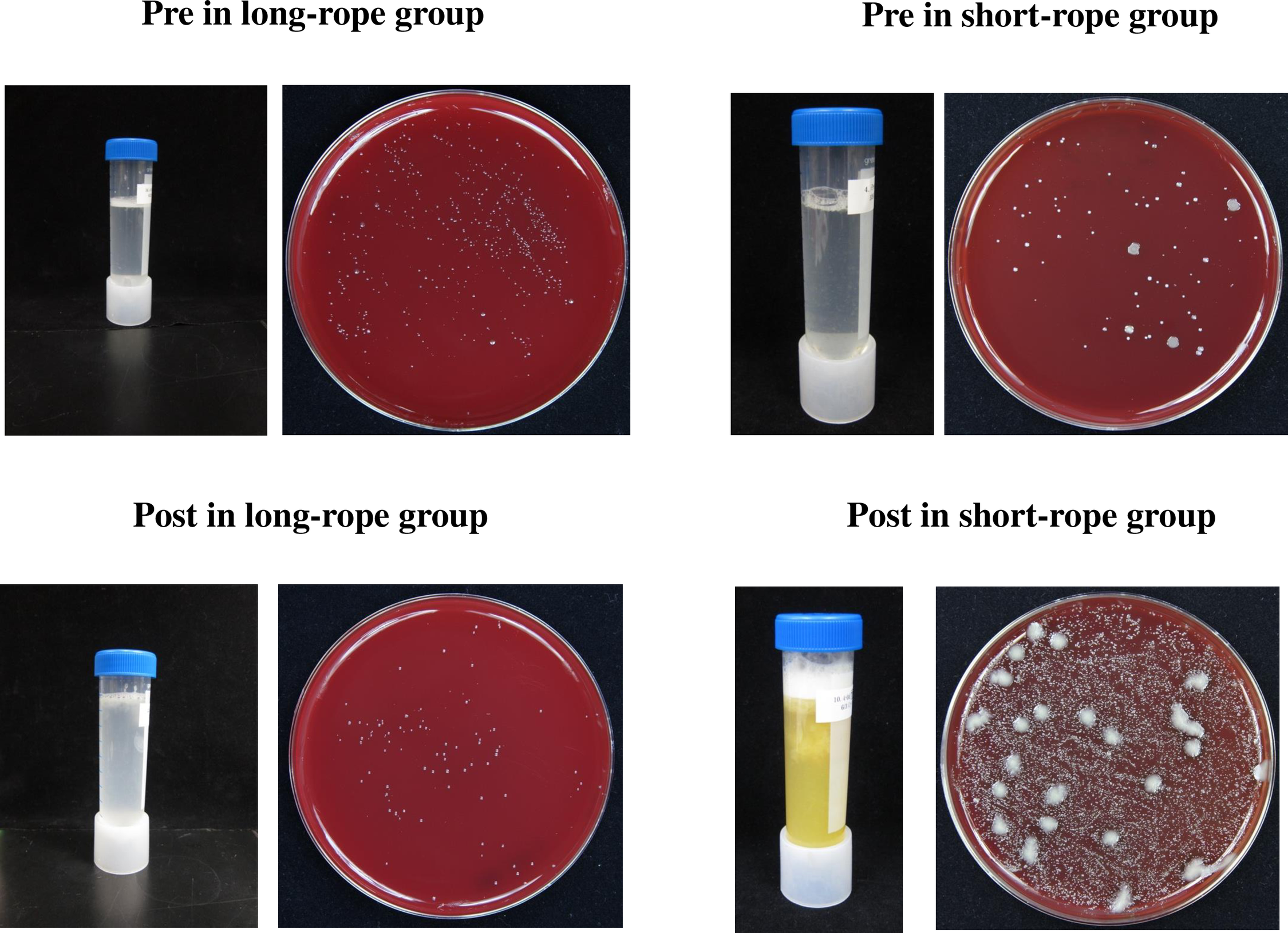


**Figure S2.** Representative images of tracheal wash and blood agar medium. These samples are from the Horse 4 shown in Table S2.


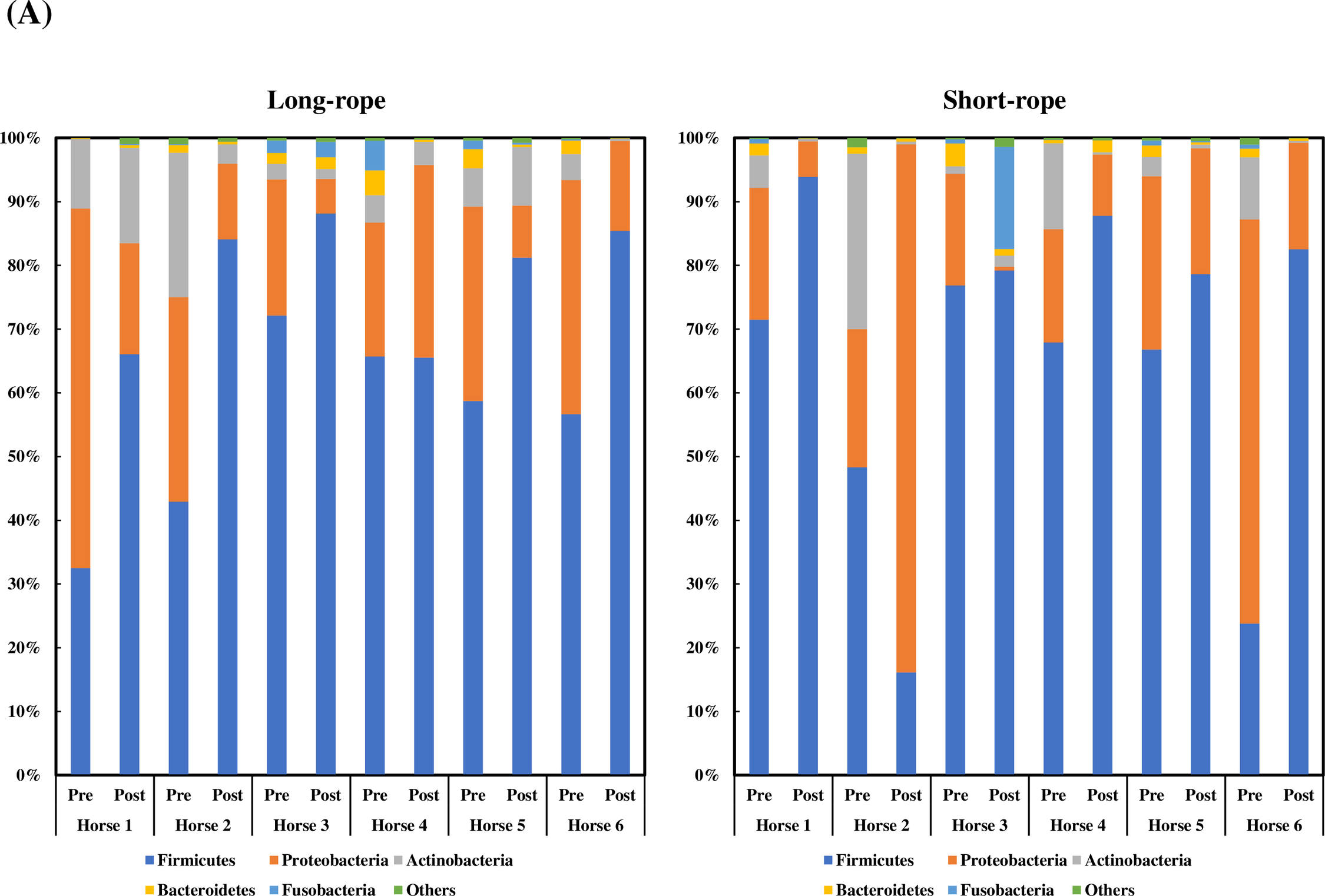


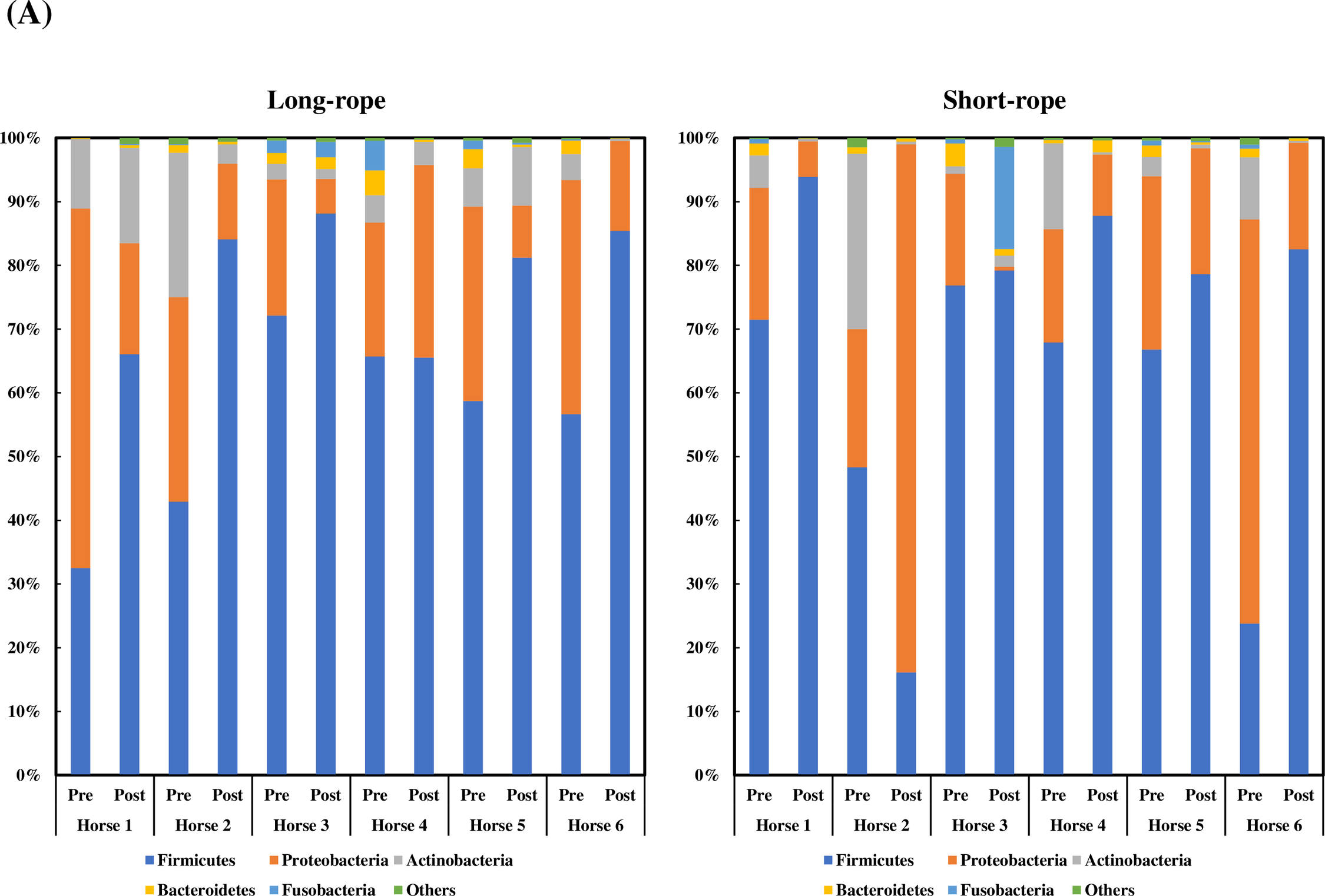


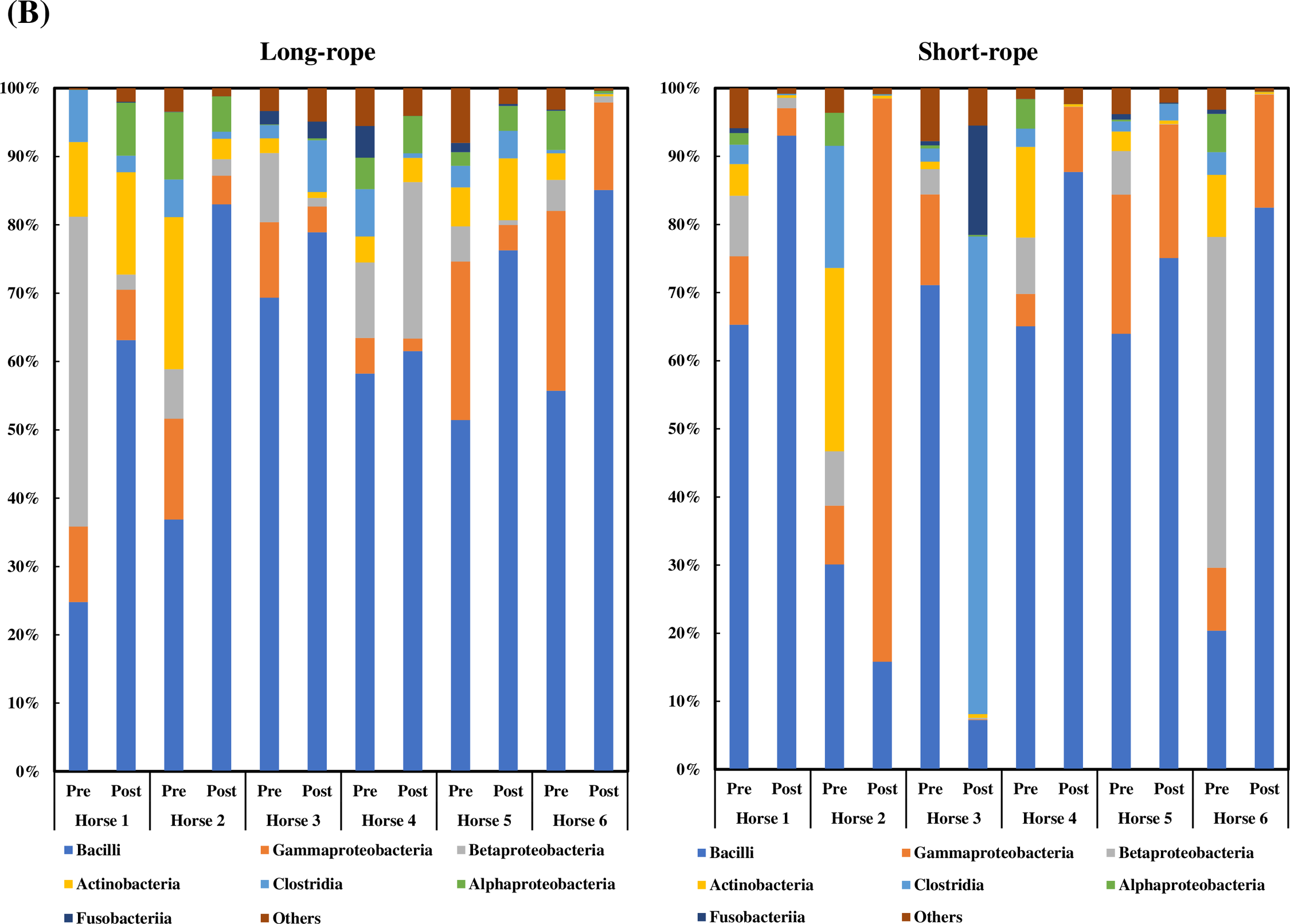


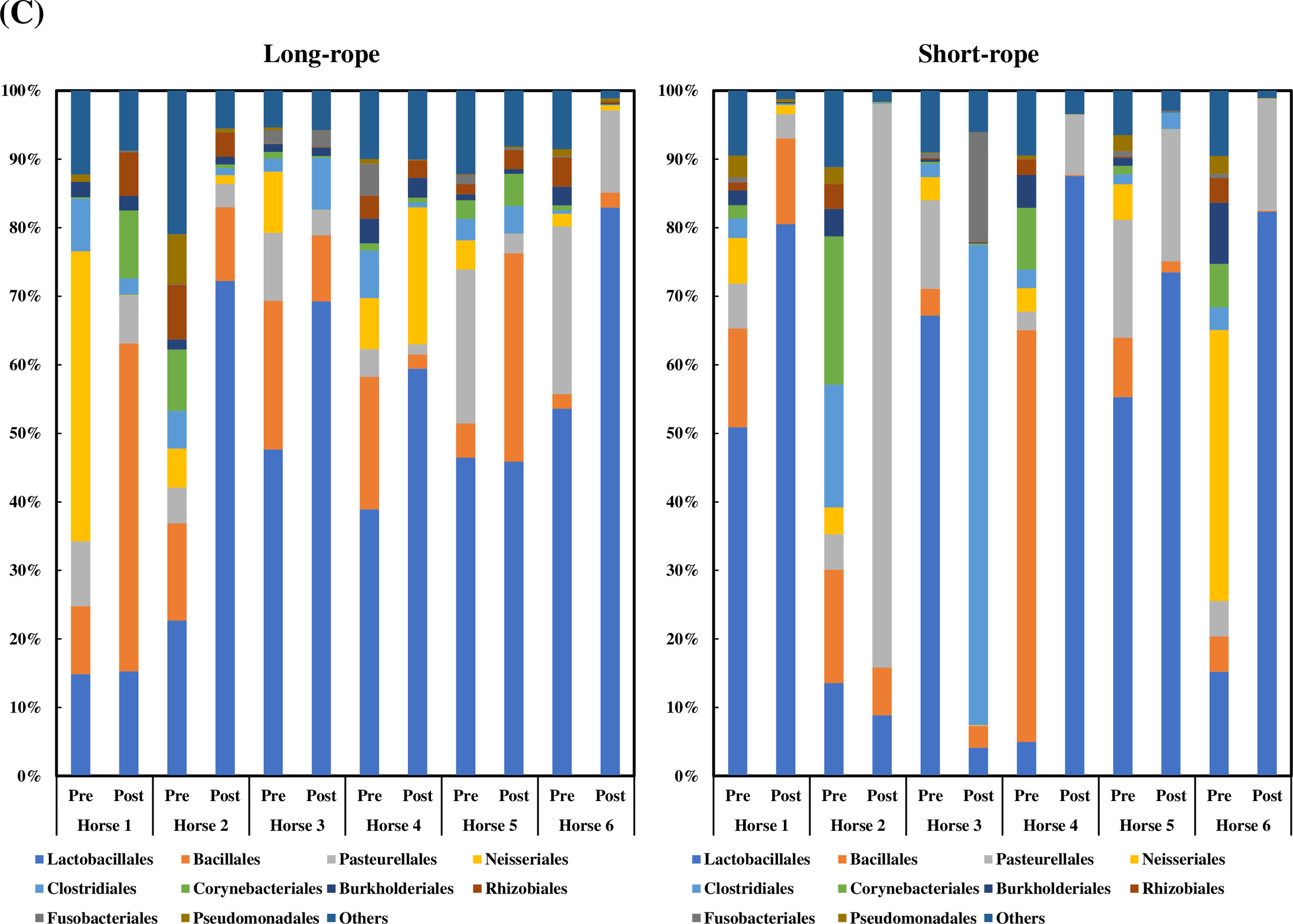


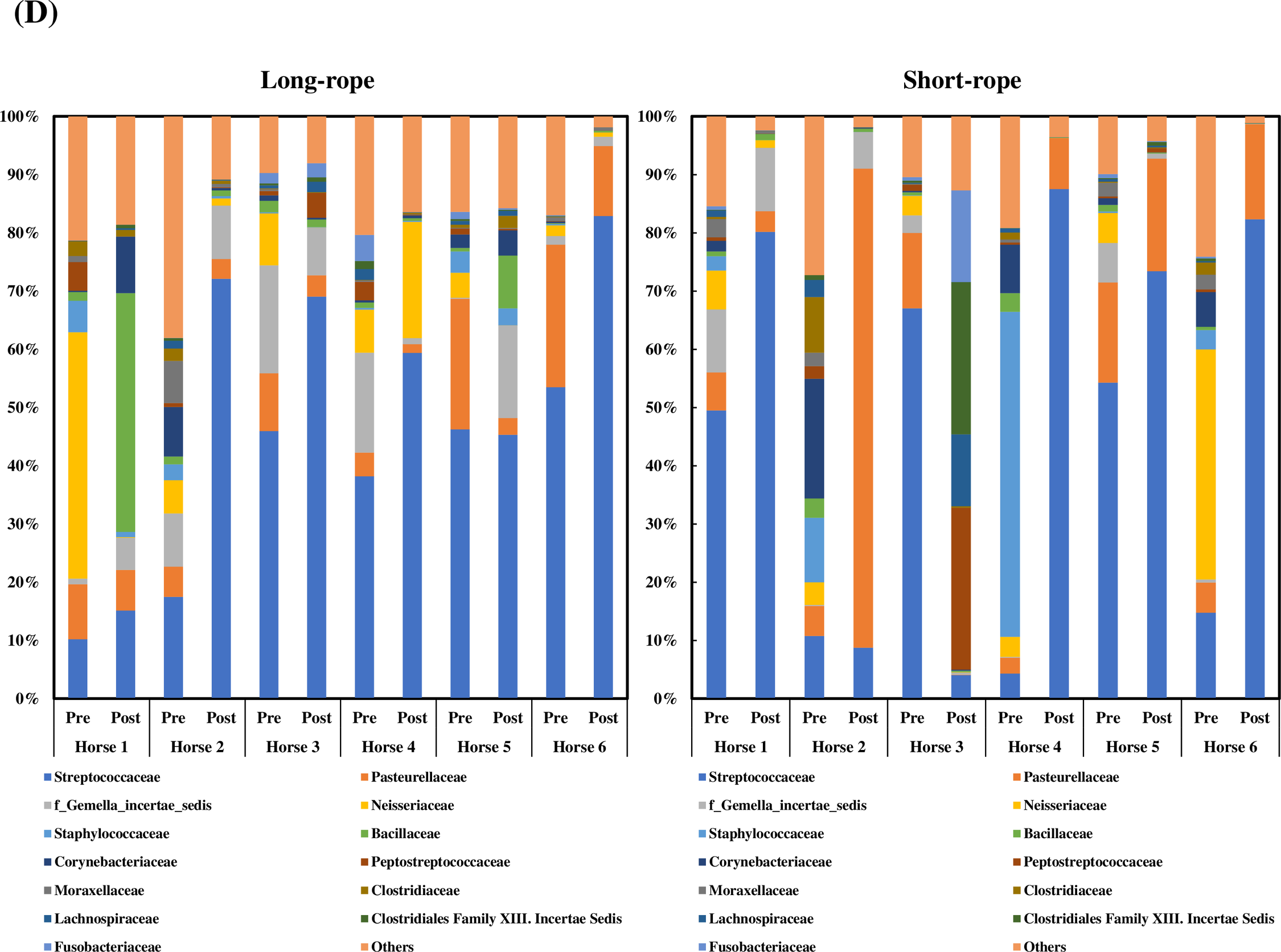


**Figure S3.** Relative abundance in tracheal wash before and after transport in each horse. (A) phylum, (B) class, (C) order, (D) family.
